# Multifunctional Crosslinked PAA–TA Binder: Robust Structural Integrity and Suppressed Manganese Dissolution for High–Performance LiMn_0.6_Fe_0.4_PO_4_ Cathodes

**DOI:** 10.3390/polym18182308

**Published:** 2026-09-21

**Authors:** Jiajun Zhou, Weibin Zhao, Jinchang Xu, Yue Li, Fenghao Zheng, Aiguo Chen, Junjie Tong, Yangxi Liu, Haoxiang Zhong

**Affiliations:** 1 School of Marine Equipment Engineering, Guangzhou Maritime University, Guangzhou 510725, China; 2 Guangdong Provincial Key Laboratory of Terahertz Quantum Electromagnetics, GBA Branch of Aerospace Information Research Institute, Chinese Academy of Sciences, Guangzhou 510700, China; 3 School of Materials and Energy, Guangdong University of Technology, Guangzhou 510006, China

**Keywords:** PAA–TA water–soluble binder, 3D crosslinked network, Mn^2+^ dissolution suppression, LiMn_0.6_Fe_0.4_PO_4_ cathode, lithium ion battery

## Abstract

A poly (acrylic acid–tannic acid) (PAA–TA) crosslinked composite is reported on for the first time as an aqueous binder for LiMn_0.6_Fe_0.4_PO_4_ (LMFP) cathodes to suppress manganese dissolution. Benefiting from hydrogen bonding and chemical crosslinking, the PAA-TA binder constructs a robust three-dimensional network that strengthens the interfacial adhesion among active particles, conductive additives, and the current collector, stabilizing the LMFP crystal structure upon cycling. More importantly, this polymeric network effectively restrains manganese leaching, which is the major origin of the capacity fading of LMFP cathodes. Meanwhile, continuous electron and ion transport pathways are well established. The LMFP electrode with an optimized PA5TA1 binder delivers a reversible capacity of 112 mAh g^−1^ at 5 C and retains 81.3% of its capacity after 300 cycles, outperforming the PVDF/LMFP electrode. Post–mortem analyses of cycled LMFP cathodes further confirm that there was substantially suppressed Mn^2+^ dissolution in the PAA–TA electrode, corroborating the structural integrity of the cathode–electrolyte interface. This crosslinked aqueous polymer provides a sustainable alternative to the PVDF binder for long–life LMFP cathodes.

## 1. Introduction

Lithium–ion batteries (LIBs) have long dominated the markets for portable electronic devices and electric vehicles. Nevertheless, there remains an urgent demand for electrode materials capable of delivering high energy density, extended cycle lifespan, and low manufacturing cost [1]. Among emerging cathode materials, lithium manganese iron phosphate (LMFP) stands out due to its high theoretical capacity, elevated operating voltage, abundant raw material reserves, and excellent structural/thermal stability, making it one of the most promising candidates for next–generation high–energy LIB cathodes [2]. However, LMFP cathodes still suffer from several intractable bottlenecks, intrinsically low electronic conductivity, the Jahn–Teller distortion triggered by Mn^3+^ ions [3,4], volume expansion/contraction during cycling, and inadequate interfacial bonding with current collectors, which lead to capacity decay, inferior Coulombic efficiency, and deteriorated cycling stability, thereby greatly hindering large–scale practical applications [5,6].

Binders, though constituting a minor fraction of the electrode, play a vital role in maintaining structural integrity, enhancing interfacial adhesion, and stabilizing electrode–electrolyte interfaces [7]. Polyvinylidene fluoride (PVDF) is the commercially dominant non–aqueous binder for electrode materials by virtue of its excellent binding capability and electrochemical stability [8,9]. Nevertheless, the PVDF/N–methyl–2–pyrrolidone (NMP) binder suffers from drawbacks, including high solvent toxicity, poor environmental compatibility, and high production costs [10,11]. Against this backdrop, considerable effort has recently been devoted to developing alternative binders for lithium–ion batteries [12,13], especially water–soluble polymers, such as polyacrylic acid (PAA), LA133, sodium alginate (SA), polyvinylpyrrolidone acrylic acid acrylamide, and sodium carboxymethyl cellulose (CMC), for cathode materials [14,15,16,17,18]. Some water–soluble binders have been reported to effectively restrain manganese dissolution and alleviate structural deterioration in manganese/LMFP cathode materials [17,18].

As one of the most widely investigated aqueous binders, PAA has attracted extensive research attention as an electrode binder on account of its robust adhesive force and favorable compatibility with water–based manufacturing routes [19]. However, pure PAA suffers from high rigidity and low ductility, rendering it incapable of accommodating the structural variation in LMFP during long–term cycling [20]. Moreover, PAA with the single carboxyl–based interaction also provides limited anchoring capacity for soluble Mn^2+^, restricting further improvement in cycling lifespan [21]. Tannic acid (TA), a low–cost and green natural polyphenol rich in dense ortho–phenolic hydroxyl groups, can construct dynamic hydrogen bond crosslinked networks with PAA chains [22,23]. The PAA–TA binder effectively compensates for the mechanical deficiencies of pure PAA, endowing the binder system with improved flexibility to accommodate structural deformation [24]. More importantly, the phenolic moieties of TA with intrinsic reducibility and radical–scavenging antioxidant activity immobilize leached manganese ions [25]. This unique polyphenol–metal chelation achieves simultaneous structural stabilization and Mn^2+^ dissolution inhibition, delivering a distinct synergistic modification effect. Despite these merits, the application of PAA–TA binders in LMFP cathodes remains unexplored, and their regulatory effects on electrode electrochemical performance and structural stability are yet to be clarified.

Herein, we report the fabrication of a hydrogen bonding and chemical crosslinked PAA–TA composite binder and its application to LMFP cathodes. The synergistic effects of mechanical flexibility and Mn^2+^ chelating immobilization derived from the PAA–TA network were systematically investigated. The structural stability, interfacial compatibility, and long–term cycling performance of LMFP electrodes with optimal PAA–TA binders were comprehensively evaluated. This work aims to provide a facile and sustainable binder strategy for high–stability manganese–containing phosphate cathode materials.

## 2. Materials and Methods

### 2.1. Materials

Poly (acrylic acid) (PAA, average Mv ~450,000, Aladdin, Shanghai, China), tannic acid (TA, 99%, Adamas–beta, Shanghai, China), commercial LiMn_0.6_Fe_0.4_PO_4_ nanoparticles (Shenzhen Dynanonic, Shenzhen, China), Super P Li (Canrd Technology, Dongguan, China), and Celgard 2400 polypropylene separator (Celgard, LLC, Charlotte, NC, USA) were used as received without further purification. The electrolyte was 1 M LiPF_6_ in a 1:1:1 (*w*/*w*/*w*) mixture of ethylene carbonate (EC), methyl ethyl carbonate (EMC), and diethyl carbonate (DEC). Lithium metal foils (thickness 250 µm, diameter 16 mm) were purchased from Canrd Technology Co., Ltd. (Dongguan, China) All other chemicals and solvents were purchased from reliable commercial suppliers and used without further treatment.

### 2.2. Synthesis of Binder

For aqueous binders, PAA (10 wt%) solution was obtained by dissolving PAA powder in deionized water at room temperature. A series of PAA–TA composite binders with different PAA/TA mass ratios (9:1, 5:1, and 3:1) was similarly prepared at a fixed total solid content of 10 wt%. Each mixture was magnetically stirred for 48 h at room temperature to form a homogeneous solution, which were denoted as PA9TA1, PA5TA1, and PA3TA1, respectively. For comparison, a 10 wt% PVDF binder solution was prepared by dissolving PVDF powder in N–methyl–2–pyrrolidone (NMP) under identical room–temperature conditions.

### 2.3. Fabrication of LiMn_0.6_Fe_0.4_PO_4_ Cathode

LiMn_0.6_Fe_0.4_PO_4_ (80 wt%) was used as the cathode active material. For the PVDF/LMFP electrode, the active material, Super P (10 wt%), and PVDF solution (10 wt%) were homogenously dispersed in NMP using a micro vibratory ball mill (MSK–SFM–12M, KEJING). For PAA and PAA–TA-based electrodes, the identical mass ratio of 80:10:10 was used, while deionized water was utilized as the dispersing medium instead of NMP. The resultant slurry was uniformly cast onto an aluminum foil current collector using a doctor blade. All wet electrode sheets were pre–dried at 70 °C in a forced–air drying oven, followed by vacuum dehydration at 150 °C for 2 h to remove residual solvent.

### 2.4. Material Characterization

The crystal structures of the samples were characterized by X–ray diffraction (XRD, Bruker D8 Advance) with Cu Kα radiation (λ = 1.5406 Å) at a scanning rate of 5° min^−1^ over a 2θ range of 10–80°. Fourier transform infrared spectroscopy (FT–IR, Bruker, Germany) was used to analyze the functional groups compositions of the different binders. A cold field emission scanning electron microscope (SEM, GIEC–SU–70, Hitachi, Tokyo, Japan) with an accelerating voltage of 2.0 kV was used to observe the micro–morphology of LMFP materials as well as electrode surface morphologies before and after cycling. Transmission electron microscopy (TEM, JEOL JEM–2100F, JEOL, Tokyo, Japan) at 200 kV was applied to characterize the microscopic morphology of LMFP materials. The surface wettability of electrode samples was evaluated by measuring the contact angle of deionized water using a contact angle goniometer (GD–901, Dongguan Jingding Instrument Equipment, Dongguan, China). The pH stability of the binder slurries was measured to evaluate Al foil corrosion behavior. Electrolyte swelling was assessed by the mass change in electrodes before and after immersion in electrolyte. Inductively coupled plasma mass spectrometry (ICP–MS, Agilent 7900, Agilent Technologies, Santa Clara, CA, USA) was used to determine Mn dissolution in the electrolyte after 500 cycles.

### 2.5. Electrochemical Measurement

CR2032 coin–type half cells were assembled in a high–purity argon glove box (Mikrouna, Shanghai, China) with strictly controlled water and oxygen levels. Celgard 2400 was the separator, metallic lithium sheets acted as the anode, and 1 M LiPF_6_ dissolved in EC/EMC/DEC (1:1:1, *w*/*w*/*w*) was employed as the electrolyte. All C–rates in this work were calculated based on a theoretical specific capacity of 170 mAh g^−1^. Galvanostatic charge/discharge tests were performed on a Neware battery tester (Shenzhen, China) within a voltage window of 2.5–4.5 V. Cyclic voltammetry (CV, 2.5–4.5 V) and electrochemical impedance spectroscopy (EIS, 0.1 Hz–100 kHz, 5 mV amplitude) were performed on a DH7003B electrochemical workstation (Donghua, Jingjiang China). Linear sweep voltammetry (LSV) was used to evaluate the electrochemical stability of the binders. To estimate the kinetics of the Li+ transport of LMFP electrodes, the constant current charge/discharge and galvanostatic intermittent titration technique (GITT) were used on a Neware CT–4008 battery tester (Shenzhen, China) within the voltage range of 2.5–4.5 V. The measurements involved a constant current pulse of 10 min followed by a 30 min relaxation period, with the pulse current set at 0.1 C. To clarify the interfacial evolution and charge transfer kinetics of LMFP electrodes at various states of charge (SOCs), in situ electrochemical impedance spectroscopy (EIS) combined with distribution of relaxation times (DRT) analysis was conducted at 0.1 C after 2–3 stabilizing cycles.

## 3. Results and Discussion

### 3.1. The Structure and Morphologies of LiMn_0.6_Fe_0.4_PO_4_

**Figure 1:**
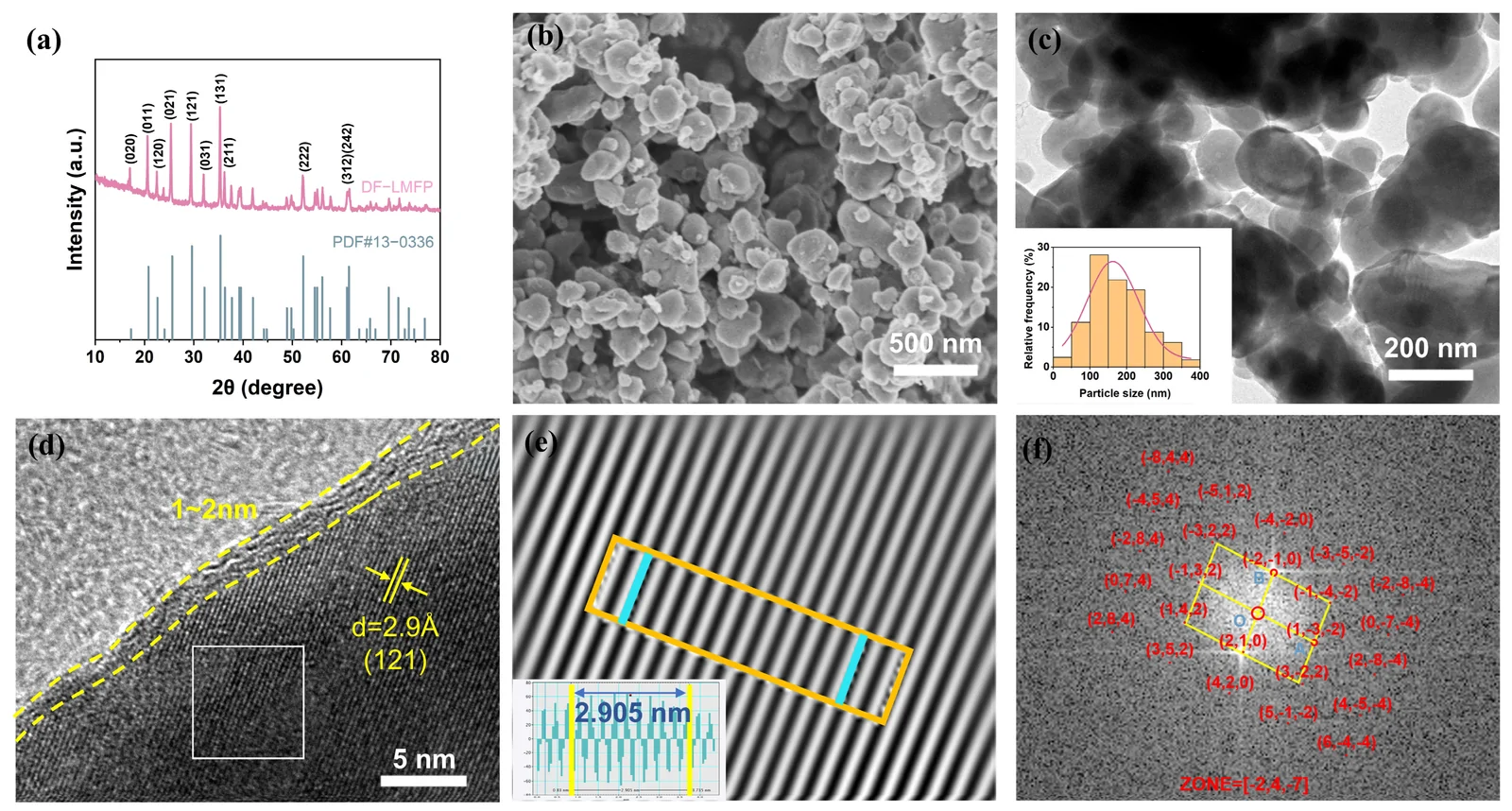
Structure characterization of LiMn_0.6_Fe_0.4_PO_4_. (**a**) XRD patterns. (**b**) SEM images. (**c**) TEM image and particle size distribution. (**d**) HRTEM images. (**e**) IFFT pattern. (**f**) SAED pattern.

XRD measurements were conducted to observe the crystalline characteristics of the commercial LiMn_0.6_Fe_0.4_PO_4_ (Figure 1a). The XRD patterns of the samples indicate that all diffraction peaks can be well–indexed to the olivine–type LiMn_0.6_Fe_0.4_PO_4_ (PDF#13–0336, orthorhombic, Pnma space group), confirming the crystal structure and high phase purity. The SEM images of LiMn_0.6_Fe_0.4_PO_4_ show irregular particles with a size distribution of 50–200 nm (Figure 1b). The TEM analysis of LiMn_0.6_Fe_0.4_PO_4_ further confirms a primary particle size of approximately 150 nm, which provides short Li^+^ diffusion pathways and enables rapid Li^+^ migration (Figure 1c). HRTEM characterization displays distinct, continuous lattice fringes with a homogeneous carbon coating with a thickness of 1–2 nm on the particle surface. The measured interplanar distance is 2.9 Å, matching the (121) crystal plane of olivine phosphate (Figure 1d). The inverse fast Fourier transform (IFFT) image derived from the selected region further eliminates background noise, and the lattice fringe spacing is precisely determined to be 2.905 Å (Figure 1e). Selected area electron diffraction (SAED) presents a series of regularly arranged sharp spots that can be well assigned to olivine LiMn_0.6_Fe_0.4_PO_4_ under the zone axis of [−2, 4, −7]. This result further verifies the high crystallinity and single−crystalline feature of the as−prepared LMFP (Figure 1f).

### 3.2. The Structure, Electrolyte Swelling, and Electrochemical Stability of the PAATA Binder

**Figure 2:**
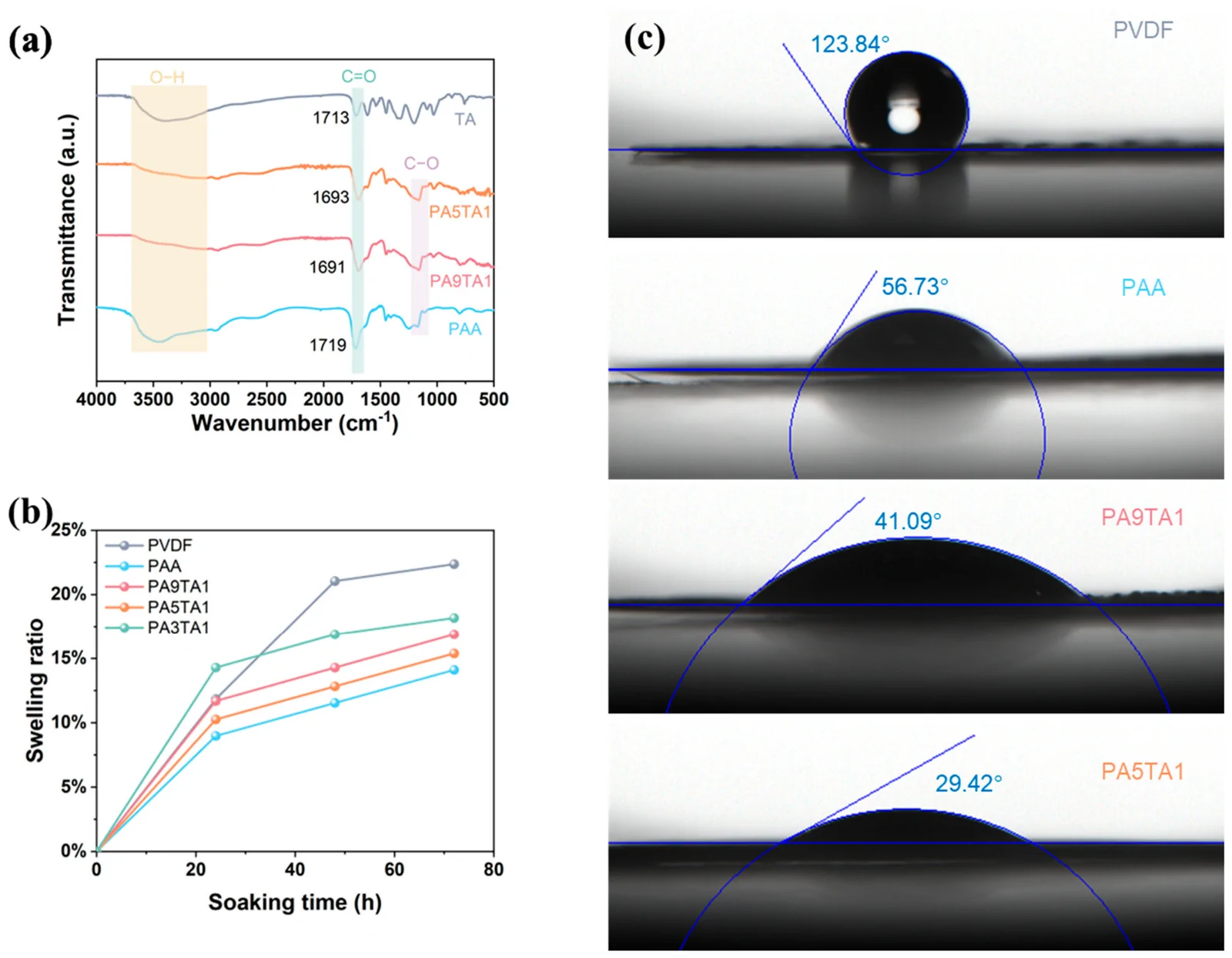
Characterizations of chemical composition and wetting properties of different binders. (**a**) FTIR spectra. (**b**) Swelling ratio of electrode sheets after soaking in electrolyte (mass change relative to dry state). (**c**) Static contact angles of water droplets on LMFP electrode sheets.

FT–IR spectroscopy was used to verify the intermolecular interactions for PAA and TA and PAATA annealed at 150 °C for 2 h (Figure 2a). The strong and broad hydroxyl absorption at 3200–3600 cm^−1^ observed for pure PAA and TA becomes extremely weak in PAATA, indicating that most hydroxyl groups are engaged in strong intermolecular interactions with carbonyl or other polar groups. The C=O peak of PAA shifts from 1719 cm^−1^ to around 1692 cm^−1^ in PAATA due to intermolecular interactions [26]. The characteristic benzene ring vibration of TA at 1613 cm^−1^ confirms the successful incorporation of TA [27], and the aliphatic C–O stretching peak shifts from 1168 cm^−1^ to 1159 cm^−1^, further evidencing the coexistence of hydrogen bonding within PAATA [28].

To further elucidate the crosslinking mechanism, XPS analysis was performed, with detailed spectra and fitting results provided in the Supporting Information (Figure S1). In the C1s spectrum, the O–C=O component (288.8 eV) decreases significantly from 30.2% in pure PAA to 11.3% in PAATA, indicating the consumption of PAA carboxyl groups. Meanwhile, a new C–O/C–OH component emerges at 286.3 eV, accounting for 18.3%, which can be attributed to the introduction of phenolic hydroxyls from TA and the C–O bonds in the newly formed ester linkages. Notably, although TA is rich in phenolic hydroxyls, the C–OH proportion in the O1s spectrum decreases from 55.3% to 49.5%. This decrease indicates the consumption of hydroxyl groups, which can be reasonably attributed to esterification with PAA carboxyl groups. Together with the reduction in O–C=O and the emergence of C–O, these results suggest the formation of a covalently crosslinked network [29]. To verify the formation of the crosslinked network, dissolution tests were conducted (Figure S2). Pristine PAA readily dissolves in water, forming a transparent solution, whereas the PAATA film remains intact without noticeable dissolution even after 48 h of immersion in water. This distinct difference in dissolution behavior supports the existence of a stable crosslinked structure in PAATA at 150 °C for 2 h.

The mass change in PAA and PAATA films after 72 h of electrolyte immersion is presented in Figure S3. PAA exhibits the lowest electrolyte uptake of 33.2%, which arises from the tightly compacted polymer chain stacking induced by extensive interchain hydrogen bonding between carboxylic groups. Upon the introduction of TA, bulky polyphenol molecules intercalate into the PAA polymer chains and expand the network structure, increasing the electrolyte uptake. For the PA5TA1 film, abundant phenolic hydroxyl groups of TA form hydrogen bonding and chemical crosslinks with the carboxyl groups of PAA. This dense and integrated crosslinked framework effectively restrains excessive electrolyte uptake, thereby limiting the mass increase to 33.5%. In contrast, PA9TA1 possesses inadequate TA to construct sufficient crosslinking sites. The discontinuous network fails to confine polymer chain movement, generating abundant internal voids that facilitate electrolyte infiltration, which ultimately yield a higher uptake of 35.3%. For PA3TA1, excessive TA content introduces abundant polar phenolic hydroxyl groups that further enhance electrolyte affinity, while the overcrowded crosslinking configuration disrupts regular chain packing, leading to the highest uptake of 37.8%. This intrinsic anti–swelling capability, together with the dissolution resistance observed in Figure S2, confirms the structural stability of the PAATA network in electrolyte environments.

Linear sweep voltammetry (LSV) was further used to evaluate the electrochemical stability of the binders. The measurements were performed over a potential window of 3.0–6.0 V, wherein the working electrode consisted of binder cast onto aluminum foil without incorporating any active materials. Only negligible oxidative current was detected at potentials below 4.5 V, which confirms that the binders possess favorable electrochemical stability between 3.0 V and 4.5 V (Figure S4).

### 3.3. pH Stability, Electrolyte Swelling, and Surface Wettability of LMFP Cathode Sheet

To mitigate the corrosion of aluminum current collectors, we monitored the pH evolution of LiMn_0_._6_Fe_0_._4_PO_4_ slurries with distinct binders. As illustrated in Figure S5, the pristine LiMn_0.6_Fe_0.4_PO_4_ slurry exhibits an initial pH of roughly 10.5, which gradually climbs to 10.7 within 12 h. Such strongly alkaline conditions exceed the pH tolerance range (4.0–8.5) of aluminum foils [30]. In sharp contrast, the PAATA binder effectively suppresses pH drift. The PA5TA1/LMFP slurry shows an initial pH of 4.22 at 1 min, followed by a slight rise to merely 5.03 after 24 h, well within the corrosion–stable pH range. All other PAATA/LMFP slurries exhibit similar pH trends and remain within the safe range. These results confirm that PAATA binders can keep slurry pH within 4.0–8.5 and effectively inhibit the corrosion of aluminum current collectors [26].

Figure 2b compares the electrolyte swelling behaviors of LMFP electrodes employing PVDF, PAA, and PAA–TA binders after 72 h of electrolyte immersion. The PVDF/LMFP, PAA/LMFP, PA9TA1/LMFP, PA5TA1/LMFP and PA3TA1/LMFP cathodes exhibit swelling ratios of 22.4%, 14.1%, 16.9%, 15.4% and 18.2%, respectively. PVDF/LMFP delivers the highest swelling ratio, whereas PAA/LMFP shows the lowest. The PA5TA1/LMFP electrode exhibits a moderate swelling ratio, as the abundant phenolic hydroxyl groups of TA form hydrogen bonds and chemical cross–links with the carboxyl groups of PAA, constructing a robust crosslinked framework that mitigates excessive electrolyte uptake.

Water contact angle measurements were performed by dropping deionized water onto the surface of as–prepared LiMn_0.6_Fe_0.4_PO_4_ electrodes with different binders, and the results are presented in Figure 2c. Lower contact angles indicate superior surface hydrophilicity, which may facilitate electrolyte infiltration into the internal pores of electrodes [31]. The PVDF/LMFP electrode sheet delivers a high contact angle of 123.8°, indicating strong hydrophobicity and poor liquid wettability. PAA shows a lower contact angle of 56.7°. With increasing TA content, the contact angle gradually decreases, demonstrating continuously improved interfacial affinity toward water. Remarkably, PA5TA1 reaches the lowest contact angle of 29.4°, greatly outperforming PVDF (123.8°), PAA (56.7°) and PA9TA1 (41.1°). This confirms that the optimized TA amount significantly improves the hydrophilicity of the binder layer, which may facilitate electrolyte impregnation inside the electrode structure.

### 3.4. The Mn Leaching Inhibition of Different Binders

Inductively coupled plasma mass spectrometry (ICP–MS) was adopted to quantitatively characterize manganese leaching from LMFP electrodes with different binders after 500 cycles. The Mn leaching concentrations were 0.076 mg/L for the PVDF/LMFP electrode, 0.049 mg/L for PA9TA1, and 0.061 mg/L for PA3TA1. By comparison, manganese was undetectable in the PA5TA1/LMFP system (<0.5 µg/L). To further investigate the migration of dissolved Mn species, we assembled a full cell using PA5TA1/LMFP as the cathode and ultra–thin Li foil (20 μm) as the anode. The separator and lithium anode were subsequently characterized by ICP–MS. For the PA5TA1–LMFP cell, the Mn concentrations detected on the separator and ultra–thin Li anodes are 0.048 mg/L and 0.053 mg/L, respectively, confirming Mn crossover originating from the PA5TA1–LMFP cathode. For comparison, the PVDF–LMFP cell exhibited Mn concentrations of 0.057 mg/L on the separator and 0.059 mg/L on the ultra–thin Li anode. These results reveal that the PA5TA1–LMFP cathode delivers suppressed Mn crossover compared with PVDF–LMFP, demonstrating the excellent capacity of the PA5TA1 binder to mitigate Mn dissolution and subsequent crossover.

The remarkable suppression of Mn leaching arises from two synergistic effects of the PA5TA1 network: chelation between polar functional groups and Mn ions and the physical confinement afforded by the crosslinked framework. On one hand, abundant carboxyl groups along PAA chains serve as anchoring sites to trap Mn^2+^ cations; on the other hand, plentiful polyphenol moieties of tannic acid form robust chelating coordination bonds with dissolved Mn^2+^ species. This dual chelation capacity of polar groups aligns with the widely reported metal–ion sequestration performance of tannic acid–containing polymeric systems [26]. Meanwhile, the dense hydrogen–bonded and covalently crosslinked network acts as a physical barrier to block the outward migration of manganese ions.

### 3.5. Electrochemical Properties of LiMn_0.6_Fe_0.4_PO_4_ Cathode

**Figure 3:**
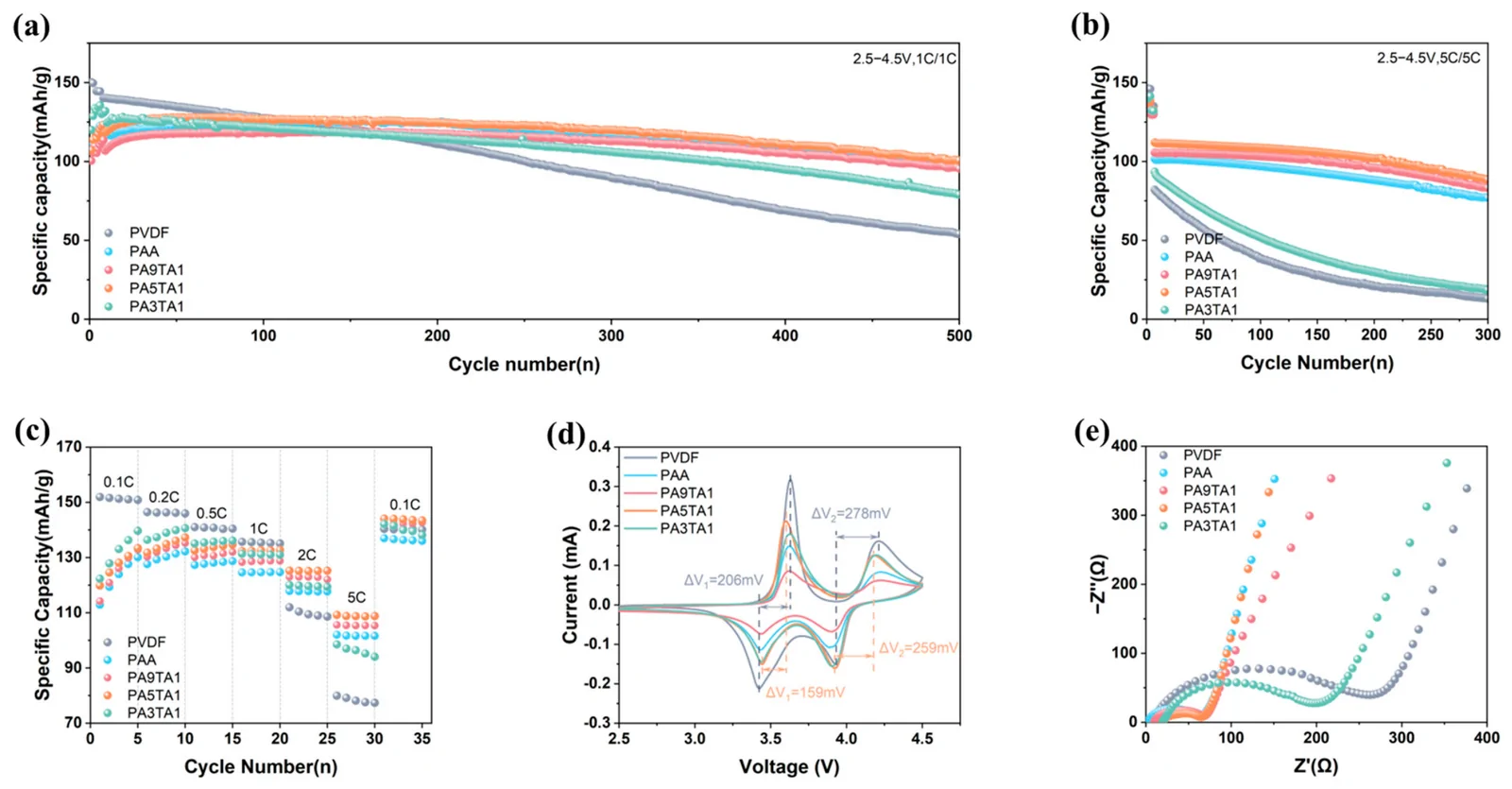
Electrochemical performance of LMFP cathodes. (**a**) Cycling performance at 1 C. (**b**) Cycling performance at 5 C/5 C. (**c**) Rate capability. (**d**) CV curves. (**e**) EIS Nyquist plots.

The long–term cycling performance of LiMn_0.6_Fe_0.4_PO_4_ electrodes with different binders at a current density of 1 C within 2.5–4.5 V is displayed in Figure 3a. Among all electrodes, the LMFP/PA5TA1 electrode exhibits the optimal cycling stability. For the water–soluble binder–based electrodes, a gradual increase in specific capacity is observed in the initial cycles, which is characteristic of the progressive electrochemical activation inherent to this binder system [32]. The LMFP/PA5TA1 electrode delivers a reversible capacity of 128.0 mAh g^−1^ and retains 93.8% capacity after 300 cycles. Even after 500 cycles, it still retains a high specific capacity of 100.8 mAh g^−1^. The overall cycling stability for all LMFP electrodes with different binders follows the following sequence: PA5TA1 > PAA > PA9TA1 > PA3TA1 > PVDF. The PAA/LMFP electrode only maintains 82.8% of its initial capacity after 300 cycles. In sharp contrast, the LMFP/PVDF electrode suffers from dramatic capacity decay. Its capacity retention drops below 80% after only 200 cycles, and the reversible capacity decays drastically to less than 60 mAh g^−1^ after 500 cycles. Meanwhile, all LMFP electrodes with the water-soluble binders exhibit much more stable coulombic efficiency than PVDF/LMFP electrodes.

To assess electrode performance under higher current density, long–term cycling tests at 5 C of LMFP electrodes with different binders were also conducted, as shown in Figure 3b. The LMFP/PA5TA1 electrode delivers a reversible capacity of 112.1 mAh g^−1^ with 81.3% capacity retention over 300 cycles, while LMFP/PA9TA1 and LMFP/PAA show comparable stability but slightly lower specific capacities. In sharp contrast, LMFP/PA3TA1 and LMFP/PVDF suffer catastrophic capacity decay, with capacity retention below 80% after only 40 cycles. The enhanced performance of the LMFP/PA5TA1 electrode is likely associated with improved interfacial kinetics and reduced Mn^2+^ dissolution.

The rate capability of LMFP electrodes with different binders was further investigated at various Crates (Figure 3c). At a low rate (0.1C–1C), the LMFP/PVDF electrode exhibits a higher specific capacity than all LMFP/PAA–TA electrodes, as electrolytes slowly infiltrate the PVDF/LMFP electrodes and gradually activate most active sites, thus yielding high specific capacity. Although they have superior intrinsic wettability, PAATA–based LMFP electrodes deliver inferior low–rate capacity mainly due to side reactions originating at the hydrophilic binder–electrolyte interface, as well as minor microstructure heterogeneity during fabrication [33].

For PAA–TA/LMFP, increasing TA content improves electrolyte wettability, shortens electrode activation time (PAA > PA9TA1 > PA5TA1 > PA3TA1), and accelerates Li^+^ transport, which is consistent with the contact angle results. However, rate performance does not improve monotonically with TA loading. PA5TA1 achieves optimal overall rate capability, while PA3TA1 performs worse despite its fastest electrolyte infiltration. Excessive TA introduces adverse effects including elevated interfacial resistance and deteriorated mechanical adhesion, which dominate under high current and counteract the wettability advantage. Therefore, moderate TA incorporation is essential.

At high rates (>2 C), electrochemical behavior is dominated by interfacial kinetics, where water–soluble PAA–TA binders show remarkable advantages over PVDF. At 5 C, LMFP/PA5TA1 retains a discharge capacity of 109 mAh g^−1^, whereas LMFP/PVDF suffers a severe capacity decay to only 78 mAh g^−1^. PA5TA1 maintains stable capacity across all tested C–rates. When the current density is switched back to 0.1C, the PA5TA1 electrode still retains a higher reversible capacity than the LMFP/PVDF electrode. This higher recovered capacity compared with the initial 0.1 C cycles is attributed to the further activation and improved interfacial contact induced by the preceding high–rate cycling, which activates additional active sites and reduces interfacial resistance.

In summary, low–rate performance is mainly limited by interfacial stability between the binder and active material, whereas high–rate performance depends on electrolyte wettability and interfacial kinetics. The optimized PA5TA1 binder network balances these factors, enabling fast interfacial kinetics and superior electrode stability under high–current conditions.

Figure 3d shows the CV profiles of all LMFP electrodes measured at a scan rate of 0.1 mV s^−1^. The redox couple at approximately 3.6 V corresponds to the Fe^3+^/Fe^2+^ couple, and that at approximately 4.1 V corresponds to the Mn^3+^/Mn^2+^ couple. The LMFP/PA5TA1 electrode exhibits the minimum ΔEp (0.16 V and 0.26 V for two couples), suggesting reduced polarization and accelerated reaction kinetics. In comparison, the LMFP/PVDF electrode shows a larger Fe ΔEp (0.21 V) and Mn ΔEp (0.28 V), which is higher than that of LMFP/PA5TA1. This result reveals more severe Fe polarization and sluggish Mn redox kinetics in the PVDF/LMFP electrode than in the PA5TA1/LMFP electrode.

Meanwhile, LMFP/PA9TA1, LMFP/PA3TA1 and LMFP/PAA show moderate values, indicating that inappropriate crosslinking aggravates polarization and impedes charge transfer kinetics.

To further analyze interfacial charge transfer kinetics, electrochemical impedance spectroscopy (EIS) was conducted under open circuit potential after the rate capability tests. The corresponding Nyquist curves are displayed in Figure 3e. EIS data were fitted using the equivalent circuit R_s_–(R_ct_//CPE)–W (Figure S6), and the fitted parameters are summarized in Table S2. The LMFP/PA5TA1, LMFP/PA9TA1, and LMFP/PAA electrodes show relatively small and comparable semicircles, indicative of low charge transfer resistance and favorable interfacial charge transport. By contrast, the LMFP/PA3TA1 electrode features a considerably enlarged semicircle, which corresponds to a substantially elevated charge transfer resistance. The elevated Rct is attributed to the over–crosslinked binder network formed by PAA and TA, which impedes interfacial ion transport and causes severe charge transfer limitations under high–rate cycling [34], consistent with its inferior performance at 5 C. Among all the LMFP cathodes, the LMFP/PVDF electrode shows the largest impedance semicircle with a substantially higher charge transfer resistance. This finding agrees well with its severe electrolyte uptake and degraded structural stability after long–term cycling. The electrochemical performance of the electrode is closely related to electrode polarization, the crosslinking network of the binder, and electrolyte swelling behavior. These observations confirm that PA5TA1 constructs a well–balanced crosslinked network. This tailored network can effectively reduce charge transfer resistance and accelerate interfacial electrochemical reaction kinetics.

### 3.6. Reaction Kinetics of LMFP Cathodes with Different Binders

**Figure 4:**
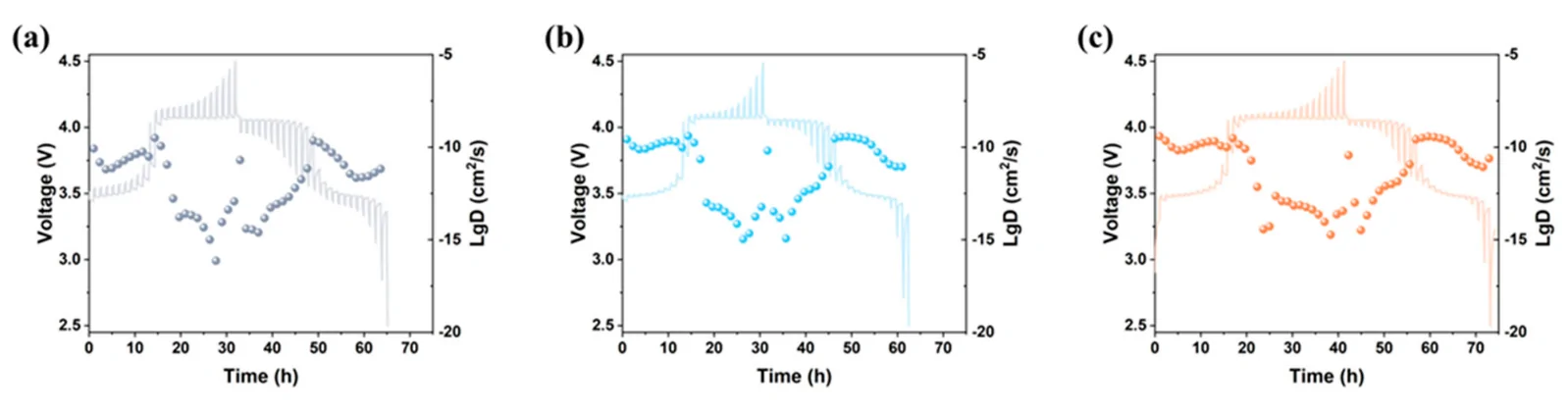
Lithium–ion diffusion coefficients of LMFP electrodes with different binders during charge–discharge processes, obtained from GITT measurements: (**a**) PVDF, (**b**) PAA, (**c**) PA5TA1.

To quantitatively evaluate lithium–ion diffusion kinetics, galvanostatic intermittent titration technique (GITT) measurements were conducted on LMFP electrodes with different binders (Figure 4). These measurements were performed after the rate capability tests. The lithium–ion diffusion coefficient was calculated according to the following equation:

(1) $$D_{{Li}^{+}}=\frac{4}{\pi \tau }{\left(\frac{m_{B}V_{M}}{M_{B}S}\right)}^{2}{\left(\frac{∆E_{S}}{∆E_{\tau }}\right)}^{2}$$

where τ is the relaxation time of the current pulse (s); *m_B_* is the mass of the electrode material; *S* is the electrode area (m^2^); *∆Eτ* and *∆Es* represent the total voltage change and the voltage change caused by the pulse, respectively; *V_M_* is the molar volume of the electrode material; and *M_B_* is the molar mass. The results reveal that the LMFP/PA5TA1 cathode exhibits the highest average *D_Li+_* value of 4.07 × 10^−12^ cm^2^/s, followed by LMFP/PAA (3.87 × 10^−12^ cm^2^/s). In contrast, the LMFP/PVDF cathode shows the lowest *D_Li+_* value of merely 1.02 × 10^−12^ cm^2^/s. The diffusion coefficient of the LMFP/PA5TA1 electrode is approximately four times that of the LMFP/PVDF electrode. A comparative log D vs. E plot for all five electrodes is provided in Figure S7. This distinct difference confirms that the optimized PAA–TA crosslinking network effectively accelerates lithium–ion migration and significantly enhances the electrochemical reaction kinetics of LMFP cathodes.

To clarify the interfacial evolution and charge transfer kinetics of LMFP electrodes with different binders at various states of charge (SOCs), in situ electrochemical impedance spectroscopy (EIS) combined with distribution of relaxation times (DRT) analysis was conducted at 0.1 C after 2–3 stabilizing cycles. DRT deconvolution was processed via DRT tools [35,36].

**Figure 5:**
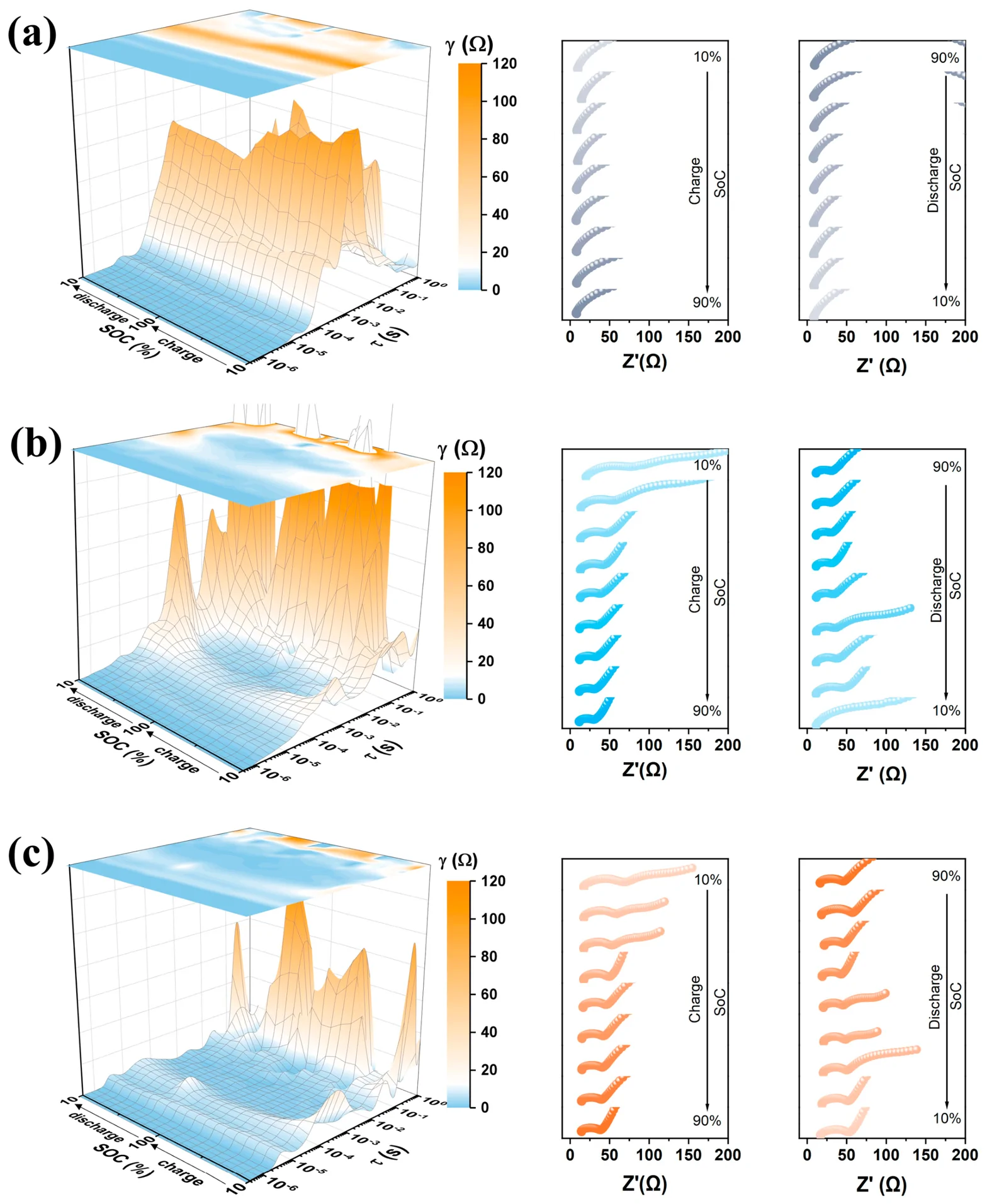
In situ DRT results of LMFP cells during cycling: (**a**) PVDF, (**b**) PAA, (**c**) PA5TA1.

Figure 5 presents the in situ DRT evolutions together with Nyquist plots for LMFP cathodes with three different binders during cycles. The 3D DRT spectra resolve three separated polarization processes, including CEI film resistance (short τ), charge transfer resistance (medium τ), and solid–state Li^+^ diffusion (long τ) [37], consistent with the capability of DRT to deconvolute interfacial film and charge transfer contributions at distinct relaxation times [38]. (a) The LMFP/PVDF electrode exhibits broad and severely overlapped DRT peaks throughout the entire SOC range, delivering the highest polarization degree. Correspondingly, it presents the largest impedance semicircle and inferior electrochemical reversibility during charge/discharge processes. (b) The LMFP/PAA cathode effectively splits the overlapping polarization peaks, reduces the overall interfacial and charge transfer resistance, and thus achieves a smaller Nyquist arc and alleviated electrochemical hysteresis compared with the LMFP/PVDF electrode. (c) The LMFP/PA5TA1 electrode exhibits the weakest DRT intensity in the medium–frequency region, indicating that the charge transfer resistance is greatly suppressed. The reduced R_CEI_ of the PA5TA1 electrode is attributed to the chelation effect of tannic acid and PAA toward Mn ions, which significantly inhibits Mn dissolution from the LMFP lattice. This mitigates continuous electrolyte decomposition and limits excessive CEI growth, thereby decreasing the migration barrier for Li^+^ transport across the CEI layer [39]. Furthermore, the preserved surface active sites and reduced surface defects optimize the charge transfer kinetics during Li^+^ intercalation/deintercalation, leading to a remarkable reduction in Rct. In comparison, pure PAA only contains carboxyl groups and possesses a much weaker metal ion chelating capacity [40]. These results confirm that the optimized PAA–TA crosslinked network effectively stabilizes the CEI film structure and substantially improves the interfacial electrochemical kinetics of LMFP cathodes, consistent with its outstanding electrochemical performance. A direct comparison of the DRT spectra for the three electrodes at selected SOCs (20%, 60%, and 100%) is provided in Figure S8, further confirming the optimized interfacial kinetics of the PA5TA1/LMFP electrode.

### 3.7. SEM Characterization of Cycled Electrodes

**Figure 6:**
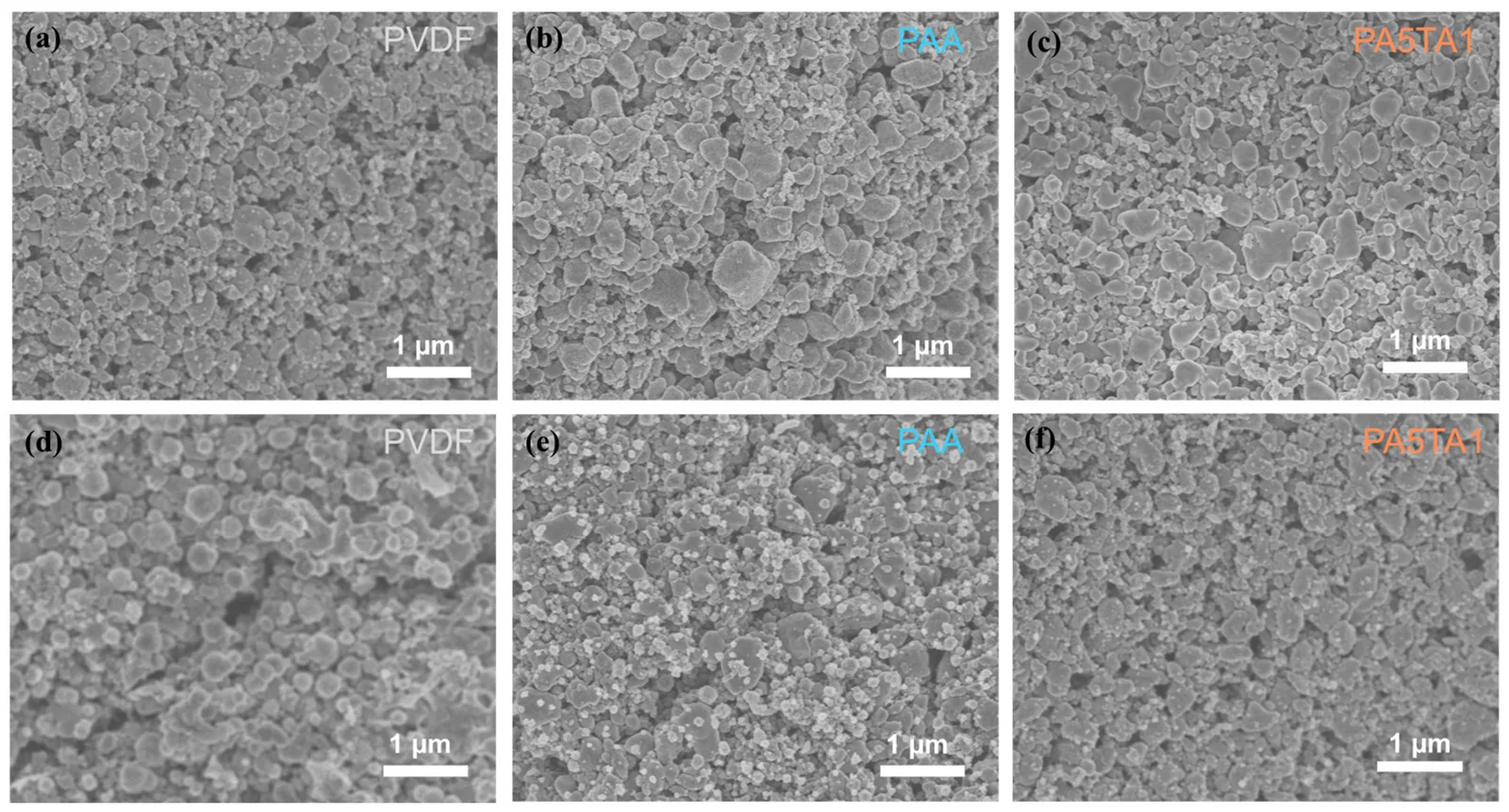
SEM images of LMFP electrodes with different binders. (**a**–**c**) Before cycling: (**a**) PVDF, (**b**) PAA, (**c**) PA5TA1. (**d**–**f**) After cycling: (**d**) PVDF, (**e**) PAA, (**f**) PA5TA1.

To investigate the structural integrity of LMFP electrodes with different binders, SEM characterizations were performed on fresh and cycled electrodes (Figure 6). All pristine electrodes show relatively smooth and intact surface morphologies. After prolonged cycling, LMFP/PVDF electrodes suffer from extensive surface cracks and severe particle pulverization owing to insufficient mechanical toughness. The LMFP/PAA electrode shows visible cracks accompanied by the partial delamination of the active material, indicating that the linear PAA polymer framework still lacks sufficient mechanical resilience. In contrast, the LMFP/PA5TA1 electrode maintains a dense, crack–free surface with well–preserved particle integrity, owing to its robust crosslinked network that effectively buffers volume variation in LMFP over repeated insertion/extraction. These results confirm that the PA5TA1 binder achieves balanced mechanical strength and flexibility, providing direct morphological evidence for the superior cycling stability of PA5TA1/LMFP electrodes.

## 4. Conclusions

In this study, an aqueous PAA–TA binder system with an optimized PAA:TA ratio of 5:1 (PA5TA1) was developed for LiMn_0.6_Fe_0.4_PO_4_ cathodes. PA5TA1 forms a crosslinked network stabilized by intermolecular hydrogen bonds and covalent chemical linkages. This elastic network accommodates volume variation in LMFP electrodes during cycling and prevents fresh active surfaces from being exposed to the electrolyte. Together with the strong chelating anchoring effect toward dissolved Mn^2+^ cations, the physical confinement provided by the crosslinked network drastically alleviates manganese dissolution. This dual–function mechanism further restrains parasitic side reactions and hinders the uncontrolled overgrowth of CEI films at the electrode–electrolyte interphase. Benefiting from the above synergistic merits, the LMFP/PA5TA1 electrode delivers outstanding cycling stability with 81.3% capacity retention after 300 cycles at 5 C and exhibits the highest Li^+^ diffusion coefficient of 4.07 × 10^−12^ cm^2^/s, moderately exceeding that of pure PAA (3.87 × 10^−12^ cm^2^/s). Post–mortem SEM and in situ DRT–EIS characterizations jointly verify that the crosslinked binder inhibits structural pulverization and stabilizes electrode–electrolyte interfaces. This study demonstrates that the rational tuning of the component ratio for dual–crosslinked polymer binders serves as a reliable strategy to suppress Mn^2+^ leaching and fabricate long–life LMFP cathodes suitable for high–power lithium–ion battery applications.

## Data Availability

The raw data supporting the conclusions of this article will be made available by the authors on request.

## Supplementary Materials

The following supporting information can be downloaded at https://www.mdpi.com/article/10.3390/polym18182308/s1. Figure S1: XPS survey and high–resolution C 1s, O 1s spectra of PAA and PA5TA1 binder films; Figure S2: Photographs of PAA, PA9TA1, PA5TA1, and PA3TA1 films after 150 °C for 2 h and 48 h water immersion; Figure S3: Electrolyte uptake of free–standing PAA–TA binder films after 72 h immersion in electrolyte; Figure S4: LSV curves of PAA and PA5TA1 binders (3.0–6.0 V) at scan rate of 2mV/s; Figure S5: pH variation in LiMn_0.6_Fe_0.4_PO_4_ slurries with different binders over 50 h; Figure S6: Equivalent circuit Rs–(Rct//CPE)–W for EIS fitting; Figure S7: Log D_Li_^+^ vs. potential curves of LMFP electrodes obtained by GITT; Figure S8: DRT spectra of electrodes at selected SOCs (20%, 60%, 100%). Table S1: Comparison of polarization potentials of LMFP electrodes with different binders; Table S2: EIS fitting parameters of LiMn_0.6_Fe_0.4_PO_4_ electrodes after rate capability tests.

## References

[1] Ngoy K.R., Lukong V.T., Yoro K.O., Makambo J.B., Chukwuati N.C., Ibegbulam C., Eterigho Ikelegbe O., Ukoba K., Jen T.C. (2025). Lithium-Ion Batteries and the Future of Sustainable Energy: A Comprehensive Review. Renew. Sustain. Energy Rev..

[2] Gonçalves J.M., Mubarac S., Silva G.T.M., Freitas B., Aguiar Neto B.G., Zanin H. (2025). Emerging Multimetal LMFP-based Cathodes for Lithium-Ion Batteries: A Review. J. Mater. Chem. A.

[3] Jeong B.J., Sung J.Y., Jiang F., Jung S.P., Lee C.W. (2024). Providing High Stability to Suppress Metal Dissolution in LiMn0.5Fe0.5PO4 Cathode Materials by Zn Doping. J. Energy Stor..

[4] Bing X., Niu S., Wang K., You J., Dou W., Wu Y., Cao M., Chen S., Yang J., Su X. (2025). Revealing the Role of Carbon Layers in Lithium Manganese Iron Phosphate Cathodes: Synergistic Enhancement of Interfacial Charge Transport and Structural Stability. ACS Appl. Energy Mater..

[5] Jung H., Oh C., Park S., An S., Bang J., Youn J., Lee J., Park J.-H., Han D. (2025). Lithium Manganese Iron Phosphate (LiMn_1-y_ Fe_y_ PO_4_) Rechargeable Batteries: Bridging Material Innovation with Practical Cell Design. Energy Mater..

[6] Lin W., Wu Y., Hu X., Yang P., Wen H., Zhao T., Wang L., Shen C. (2025). Achieving Fast Mn Redox Kinetics with Solvothermal Synthesized (010) Facet Preferential LiMn_0.5_ Fe_0.5_ PO_4_ Nanoplates for Li-Ion Batteries. Adv. Sustain. Syst..

[7] Lai T., Wang L., Liu Z., Bhayo A.M., Wang Y., He X. (2026). Tackling Challenges and Exploring Opportunities in Cathode Binder Innovation. Nano-Micro Lett..

[8] Mun S.C., Jeon Y.H., Won J.H. (2024). Progress and Challenges for Replacing N-Methyl-2-Pyrrolidone/Polyvinylidene Fluoride Slurry Formulations in Lithium-Ion Battery Cathodes. Prog. Nat. Sci. Mater. Int..

[9] Shin W., Kwon D., Kim M., Woo J., Jung H., Bang J., Shim J., Yoo J. (2026). Fluorine-Free Binder Strategies for High-Nickel Cathodes: Toward PFAS-Free Lithium-Ion Batteries. ChemSusChem.

[10] Tian Y., Xie J., Tian M., Luo X., Wang L., Zhou S., Feng Y., Hu L. (2025). Advanced Cathode Binders for Lithium-Ion Batteries: Molecular Design and Performance Enhancement. Chem. Eng. J. Adv..

[11] Sliz R., Valikangas J., Silva Santos H., Vilmi P., Rieppo L., Hu T., Lassi U., Fabritius T. (2022). Suitable Cathode NMP Replacement for Efficient Sustainable Printed Li-Ion Batteries. ACS Appl. Energy Mater..

[12] Nowak A.P., Trzciński K., Zarach Z., Li J., Roda D., Szkoda M. (2022). Poly (hydroxybutyrate-co-hydroxyvalerate) as a Biodegradable Binder in a Negative Electrode Material for Lithium-Ion Batteries. Appl. Surf. Sci..

[13] Dobryden I., Montanari C., Bhattacharjya D., Aydin J., Ahniyaz A. (2023). Bio-Based Binder Development for Lithium-Ion Batteries. Materials.

[14] Pan Y., Wu X., Yue T., Zhou T., Gao P., Gao M., Lei T. (2025). Novel Copolymer Dispersant with Promoted Dispersibility of the LMFP Cathode Slurry for Boosting the Li-Ion Battery Performance. ACS Appl. Energy Mater..

[15] Li J., Xiang M., Wang Y., Wu J., Zhao H., Liu H. (2017). Effects of Adhesives on the Electrochemical Performance of Monodisperse LiMn_0.8_ Fe_0.2_ PO_4_/C Microspheres as Cathode Materials for High Power Lithium-Ion Batteries. J. Mater. Chem. A.

[16] Su Y., Chen G., Chen L., Lu Y., Zhang Q., Lv Z., Li C., Li L., Liu N., Tan G. (2019). High-Rate Structure-Gradient Ni-Rich Cathode Material for Lithium-Ion Batteries. ACS Appl. Mater. Interfaces.

[17] Oishi A., Tatara R., Togo E., Inoue H., Yasuno S., Komaba S. (2022). Sulfated Alginate as an Effective Polymer Binder for High-Voltage LiNi_0.5_ Mn_1.5_ O_4_ Electrodes in Lithium-Ion Batteries. ACS Appl. Mater. Interfaces.

[18] Rao L., Jiao X., Yu C.Y., Schmidt A., O’Meara C., Seidt J., Sayre J.R., Khalifa Y.M., Kim J.H. (2022). Multifunctional Composite Binder for Thick High-Voltage Cathodes in Lithium-Ion Batteries. ACS Appl. Mater. Interfaces.

[19] Duan Y., Zeng W., Liu G., Gao L., Huang T., Peng G., Liang X., He Z., He H. (2026). PAA/HNBR Composite Binder: A Sustainable Fluorine-Free Binder Strategy for High-Rate and Durable LiFePO_4_ Cathodes. J. Power Sources.

[20] Jeong Y.H., Won G., Kim S., Jo M.S., Seo S., Jeong D., Shim J. (2025). A Tough, Adhesive, and Protective Binder Shield for Stabilizing High-Nickel Cathodes in Lithium-Ion Batteries. Adv. Energy Mater..

[21] Shen C., Yu H., Xue Z., Hu G., Cao Y., Peng Z., Du K. (2024). Bifunctional Low Molecular Weight Polyacrylic Acid as Aqueous Binder and Coating Agent Enables High Performance LiMn_2_O_4_ Cathode for Lithium-Ion Batteries. Chem. Eng. J..

[22] Zhang X., Ma Y., Feng P. (2025). Highly Sensitive and Oxidation Resistance Polyacrylic/Polyacrylamide Hydrogel for Multifunctional Flexible Sensors. ACS Appl. Polym. Mater..

[23] Tang W., Feng L., Wei X., Lai G., Chen H., Li Z., Huang X., Wu S., Lin Z. (2022). Three-Dimensional Crosslinked PAA-TA Hybrid Binders for Long-Cycle-Life SiOₓ Anodes in Lithium-Ion Batteries. ACS Appl. Mater. Interfaces.

[24] Chen J., Li Y., Wu X., Min H., Wang J., Liu X., Yang H. (2024). Dynamic Hydrogen Bond Cross-Linking Binder with Self-Healing Chemistry Enables High-Performance Silicon Anode in Lithium-Ion Batteries. J. Colloid Interface Sci..

[25] Paik S., Choi I., Lee S., Nam K.W. (2024). Chelating Effects of Polyphenolic Biomolecules to Improve β-MnO_2_ Cathode Performance for Aqueous Rechargeable Zinc-Ion Batteries. ACS Appl. Mater. Interfaces.

[26] Chunhakowit P., Phabjanda Y., Aunwisat A., Busayaporn W., Songsrirote K., Prayongpan P. (2024). Fabrication of Tannic Acid-Incorporated Polyvinylpyrrolidone/Polyvinyl Alcohol Composite Hydrogel and Its Application as an Adsorbent for Copper Ion Removal. Sci. Rep..

[27] Espina A., Sanchez-Cortes S., Jurašeková Z. (2022). Vibrational Study (Raman, SERS, and IR) of Plant Gallnut Polyphenols Related to the Fabrication of Iron Gall Inks. Molecules.

[28] Chen W., Huang W., Gupta R., Heydari A., Bagchi B., Xu L., Xue Y., Velliou E., K Tiwari M. (2026). Transparent Multifunctional Wearable Strain Sensor With Self-Healing and Antibacterial Capabilities for Human Motion Detection. Adv. Healthc. Mater..

[29] He J., Das C., Yang F., Maibach J. (2022). Crosslinked Poly(acrylic acid) Enhances Adhesion and Electrochemical Performance of Si Anodes in Li-ion Batteries. Electrochim. Acta.

[30] Doberdò I., Löffler N., Laszczynski N., Cericola D., Penazzi N., Bodoardo S., Kim G.-T., Passerini S. (2014). Enabling Aqueous Binders for Lithium Battery Cathodes–Carbon Coating of Aluminum Current Collector. J. Power Sources.

[31] Kareeklin N., Phukhrongthung A., Krittayavathananon A., Wang B., Luanwuthi S., Iamprasertkun P. (2026). Binder Chemistry of Na_3_ V_2_ (PO_4_)_2_ F_3_ Cathodes in Aqueous Sodium-Ion Batteries: From “Salt-in-Water” to “Water-in-Salt”. ACS Appl. Polym. Mater..

[32] Li J., Yang K., Zheng Y., Gao S., Chai J., Lei X., Zhan Z., Xu Y., Chen M., Liu Z. (2023). Water-soluble polyamide acid binder with fast Li+ transfer kinetics for silicon suboxide anodes in lithium-ion batteries. ACS Appl. Mater. Interfaces.

[33] Wang Y., Fang Y., Huang L., Wang J., Zhou H., Wang G., Wu Q., Wang G., Zhu H. (2025). Stabilizing Nickel-Rich Cathodes in Aqueous Process through Nanocellulose as Water Barrier. Adv. Funct. Mater..

[34] Neumann N., Abels G., Koschek K., Boskamp L. (2023). Crosslinked hyperbranched polyglycerol-based polymer electrolytes for lithium metal batteries. Batteries.

[35] Wang Z., Wang Y., Py B., Maradesa A., Liu J., Wan T.H., Saccoccio M., Ciucci F. (2025). DRTtools: Freely Accessible Distribution of Relaxation Times Analysis for Electrochemical Impedance Spectroscopy. ACS Electrochem..

[36] Chen J., Quattrocchi E., Ciucci F., Chen Y. (2023). Charging Processes in Lithium-Oxygen Batteries Unraveled through the Lens of the Distribution of Relaxation Times. Chem.

[37] Lu Y., Zhao C.-Z., Huang J.-Q., Zhang Q. (2022). The Timescale Identification Decoupling Complicated Kinetic Processes in Lithium Batteries. Joule.

[38] Geng K., Tappan B.A., Passerini S., Shao-Horn Y., Bresser D. (2026). Determination of the Exchange Current Density at Lithium │ Polymer Electrolyte Interfaces. Adv. Sci..

[39] Ou W., Marks S.D., de Menezes R.F., He R., Zhang Z., Sindt C., Thurston J., Jaye C., Cowie B., Thomsen L. (2025). Unveiling the Mec-based hanism of Mn Dissolution Through a Dynamic Cathode-Electrolyte Interphase on LiMn_2_O_4_. Adv. Energy Mater..

[40] Ciobanu R., Bucatariu F., Mihai M., Teodosiu C. (2024). SilicaComposite Sorbents for Heavy Metal Ions Removal from Aqueous Solutions. Polymers.

