# Supplementary material for: Multifunctional Crosslinked PAA–TA Binder: Robust Structural Integrity and Suppressed Manganese Dissolution for High–Performance LiMn0.6Fe0.4PO4 Cathodes
Source: Polymers (Basel). 2026 Sep 21;18(18):2308. doi: 10.3390/polym18182308 (PMC13611709; doi:10.3390/polym18182308)
Supplement: Supplementary file 1 [file polymers-18-02308-s001.zip › polymers-4457733-supplementary.pdf]

Supplementary Information

Multifunctional Crosslinked PAA−TA Binder: Robust Structural Integrity and Suppressed Manganese Dissolution for High−Performance $LiMn_{0.6}Fe_{0.4}PO_4$ Cathodes

Jiajun Zhou [1,†], Weibin Zhao [1,†], Jinchang Xu [2], Yue Li [1,3], Fenghao Zheng [1], Aiguo Chen [1], Junjie Tong [1], Yangxi Liu [1] and Haoxiang Zhong [1,*]

[1] School of Marine Equipment Engineering, Guangzhou Maritime University, Guangzhou 510725, China
[2] Guangdong Provincial Key Laboratory of Terahertz Quantum Electromagnetics, GBA Branch of Aerospace Information Research Institute, Chinese Academy of Sciences, Guangzhou 510700, China
[3] School of Materials and Energy, Guangdong University of Technology, Guangzhou 510006, China
[*] Correspondence: zhonghaoxiang@gzmtu.edu.cn
[†] These authors contributed equally to this work.

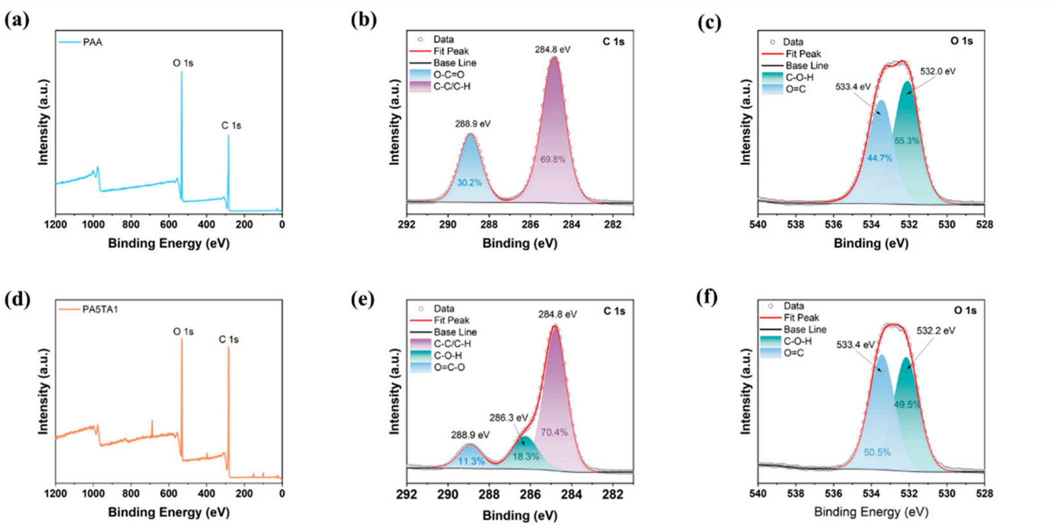

Figure S1. XPS spectra of PAA and PA5TA1. (a, d) Survey spectra of PAA and PA5TA1, respectively. (b, e) High-resolution C 1s spectra of PAA and PA5TA1, respectively. (c, f) High−resolution O 1s spectra of PAA and PA5TA1, respectively.

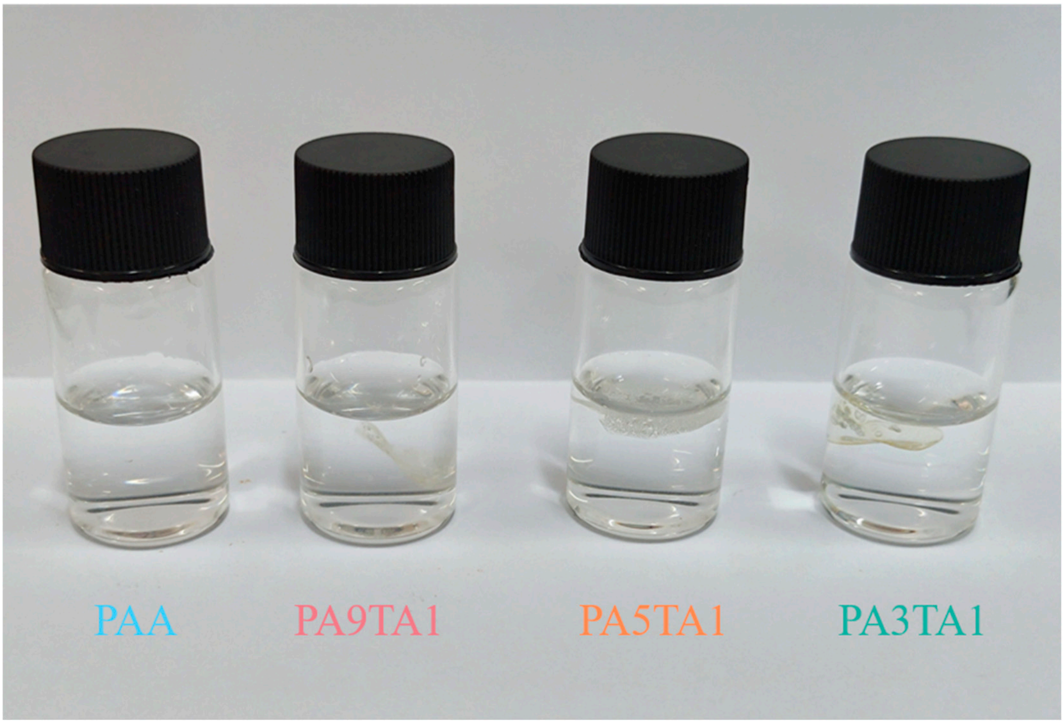

Figure S2. Photographs of PAA, PA9TA1, PA5TA1, and PA3TA1 at 150 °C for 2 h after 48 h water immersion

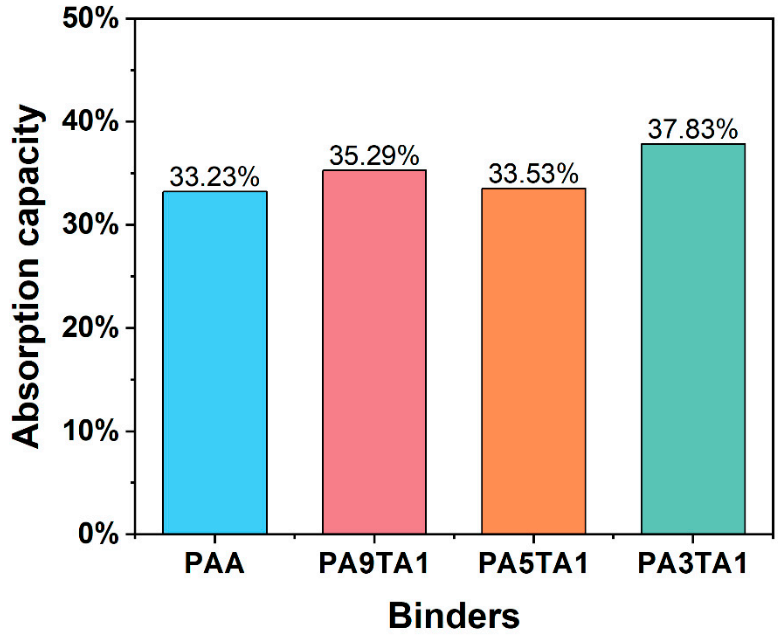

Figure S3. Electrolyte uptake of free−standing PAA and PAATA films after 72 h of immersion.

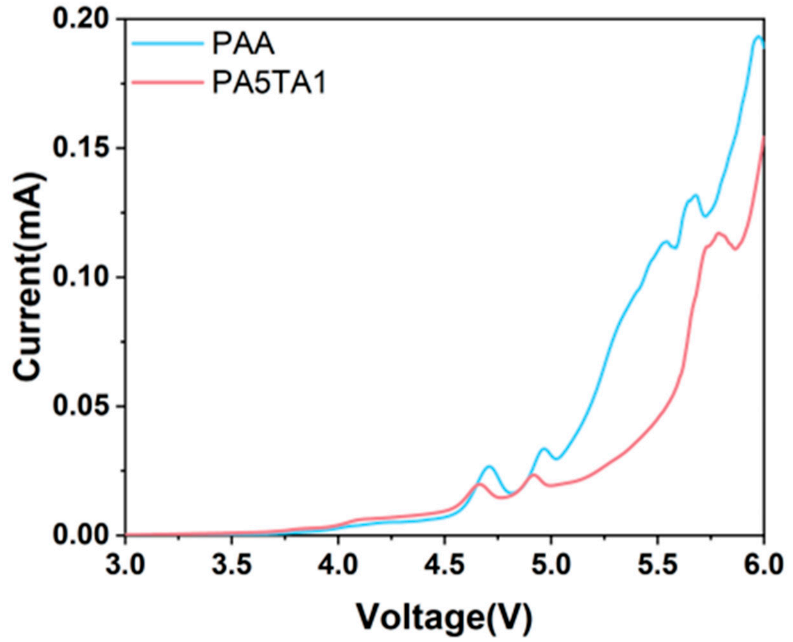

Figure S4. LSV curves of PAA and PA5TA1 binders in 3.0–6.0 V at a scan rate of 2 mV/s.

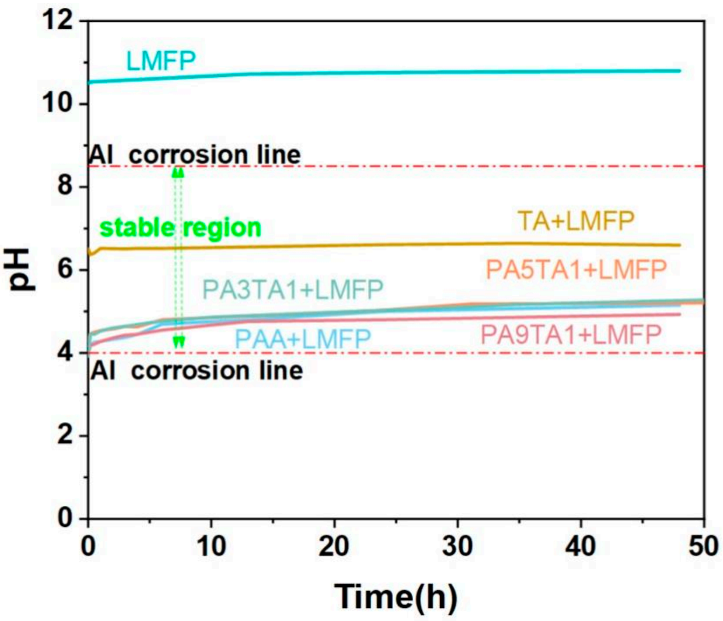

Figure S5. pH variation of $LiMn_{0.6}Fe_{0.4}PO_4$ slurries with different binders (PVDF, PAA, PA9TA1, PA5TA1, and PA3TA1) as a function of standing time over 50 h.

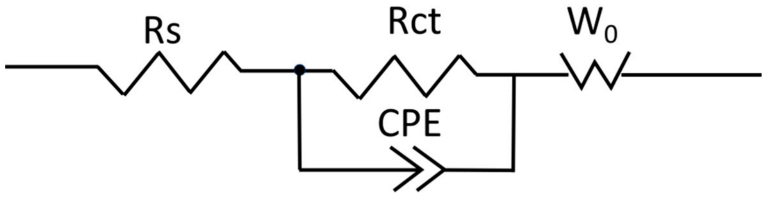

Figure S6. Equivalent circuit R_s−(R_{ct}//CPE)−W for EIS fitting.

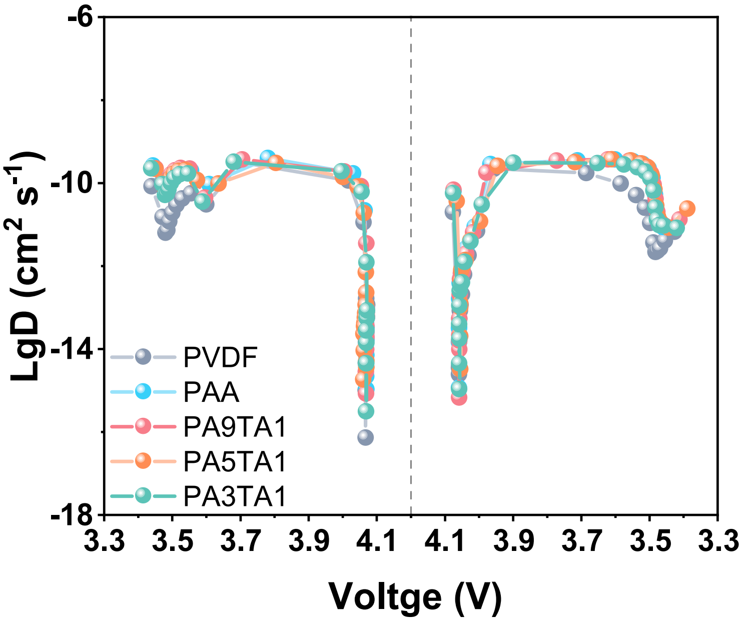

Figure S7. Log *D* vs. *E* curves of LMFP electrodes with PVDF, PAA, PA9TA1, PA5TA1, and

PA3TA1 binders.

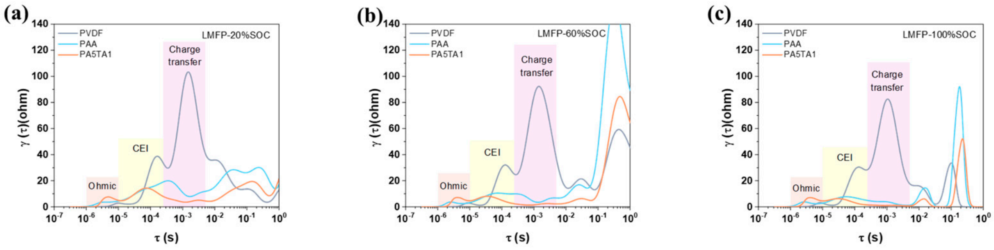

Figure S8. DRT spectra of the three electrodes at selected SOCs: (a) 20%, (b) 60%, and (c) 100%.

Table S1. Comparison of polarization potentials of LMFP electrodes with different binders.

Binders	Iron oxidation peak	Iron reduction peak	Iron polarization potential	Manganese oxidation peak	Manganese reduction peak	Manganese polarization potential
PVDF	3.6304 V	3.4241 V	0.2063 V	4.2132 V	3.9352 V	0.2780 V
PAA	3.6243 V	3.4333 V	0.1910 V	4.2261 V	3.9030 V	0.3231 V
PA9TA1	3.6215 V	3.4376 V	0.1839 V	4.2227 V	3.9023 V	0.3204 V
PA5TA1	3.6021 V	3.4428 V	0.1593 V	4.1843 V	3.9248 V	0.2595 V
PA3TA1	3.6249 V	3.4415 V	0.1834 V	4.1967 V	3.9013 V	0.2954 V

Table S2. EIS fitting parameters of $LiMn_{0.6}Fe_{0.4}PO_4$ electrodes with different binders after rate capability tests.

Binder	Rs (Ω) individual cells	Rs ± SD (Ω)	Rct (Ω) individual cells	Rct ± SD (Ω)	W ($\Omega \cdot s^{-0.5}$)
PVDF	9.4, 6.2, 7.3	7.62 ± 1.61	187.8, 170.3, 214	190.70 ± 21.99	239
PAA	4.7,4.1,2.0	3.62 ± 1.43	59.4, 37.5, 51.2	49.36 ± 11.08	39.12
PA9TA1	8.7, 10.2, 8.7	9.20 ± 0.85	53.9, 78.0, 51.0	60.99 ± 14.84	27.7
PA5TA1	19.9 17.4, 18.2	18.49 ± 1.27	43.5, 34.6, 42.8	40.28 ± 4.97	32.28
PA3TA1	16.3, 20.6, 18.9	18.60 ± 2.17	146.3, 131, 150	142.43 ± 10.07	155
